# Unlocking the Interactions Between the Whole-Body Microbiome and HPV Infection: A Literature Review

**DOI:** 10.3390/pathogens14030293

**Published:** 2025-03-18

**Authors:** Myrto Papamentzelopoulou, Vassiliki C. Pitiriga

**Affiliations:** 1 Molecular Biology Unit, 1st Department of Obstetrics and Gynecology, National and Kapodistrian University of Athens, 11527 Athens, Greece; mpntua@yahoo.gr; 2 Department of Microbiology, Medical School, National and Kapodistrian University of Athens, 75 Mikras Asias Street, 11527 Athens, Greece

**Keywords:** whole-body microbiome, human microbiome, HPV infection, human papilloma virus, probiotics, prebiotics, vaginal microbiota transplantation, personalized medicine

## Abstract

The human microbiome plays a vital role in maintaining human homeostasis, acting as a key regulator of host immunity and defense mechanisms. However, dysbiotic microbial communities may cause disruption of the symbiotic relationship between the host and the local microbiota, leading to the pathogenesis of various diseases, including viral infections and cancers. One of the most common infectious agents causing cancer is the human papilloma virus (HPV), which accounts for more than 90% of cervical cancers. In most cases, the host immune system is activated and clears HPV, whereas in some cases, the infection persists and can lead to precancerous lesions. Over the last two decades, the advent of next-generation sequencing (NGS) technology and bioinformatics has allowed a thorough and in-depth analysis of the microbial composition in various anatomical niches, allowing researchers to unveil the interactions and the underlying mechanisms through which the human microbiota could affect HPV infection establishment, persistence, and progression. Accordingly, the present narrative review aims to shed light on our understanding of the role of the human microbiome in the context of HPV infection and its progression, mainly to cervical cancer. Furthermore, we explore the mechanisms by which the composition and balance of microbial communities exert potential pathogenic or protective effects, leading to either HPV persistence and disease outcomes or clearance. Special interest is given to how the microbiome can modulate host immunity to HPV infection. Lastly, we summarize the latest findings on the therapeutic efficacy of probiotics and prebiotics in preventing and/or treating HPV infections and the potential of vaginal microbiota transplantation while highlighting the significance of personalized medicine approaches emerging from NGS-based microbiome profiling and artificial intelligence (AI) for the optimal management of HPV-related diseases.

## 1. Introduction

Virus-mediated infections are known risk factors for cancer, with human papilloma virus (HPV) being one of the most common infectious agents causing cancer (31.1%), and accounting for 4.5% of cancers globally. HPV is considered responsible for more than 90% of anal and cervical cancers, about 70% of vaginal and vulvar cancers, and more than 60% of penile cancers. Notably, recent studies have revealed that about 70% of oropharyngeal cancers may be linked to HPV infection. HPV is mainly transmitted through sexual activity, but skin-to-skin contact can also cause HPV infection. The vast majority of both men and women, approximately 80%, will be exposed to HPV at some point in their lives [1,2,3].

Papillomaviruses belong to the Papovaviridae family. HPV is a relatively small, non-enveloped virus that consists of a single molecule of double-stranded, circular DNA containing approximately 7900 bp, one regulatory region, and two early (E) and late (L) ORFs. As a tissue-specific virus, HPV affects both the cutaneous and mucosal epithelium. More than 200 HPV types have been identified and divided into low- and high-risk types. At least 14 high-risk, oncogenic HPV types are highly associated with cancer of the cervix, oropharynx, anus, vagina, vulva, and penis. The most common high-risk HPV types are 16 and 18, but other less prevalent types include 31, 33, 45, 52, and 58 [4]. The high-risk HPV types encode two oncoproteins, E6 and E7, which are required for the development and maintenance of HPV-associated cancers. The HPV replication cycle is regulated by the viral E2 protein, which serves either as a transcriptional activator or as a suppressor of viral gene expression. The promoters for E6/E7 gene expression of HPV16 and HPV18 are negatively regulated by E2 [5].

Approximately 90–95% of cervical and anal cancers, 70% of vaginal and vulvar cancers, and 60% of oropharyngeal and penile cancer cases are associated with high-risk HPV. On the other hand, low-risk HPV types, also called non-oncogenic, are associated with genital warts, respiratory tract papillomas, and low-grade abnormalities within cervical cells. The most common low-risk HPV types are 6 and 11 [4,6]. In most cases, the immune system is activated and clears the virus, whereas in some cases, the infection persists and can lead to precancerous changes. HPV type 16 has the lowest clearance rate among high-risk HPV types. Moreover, HPV infection can be influenced by factors that have significant implications for infection dynamics and disease progression. Such factors include long-term use of oral contraceptives, smoking, many pregnancies, a compromised immune system, interaction with viruses like herpes simplex virus type 1/2 (HSV1/2), cytomegalovirus (CMV), human herpesvirus 6 or 7 (HHV-6 or 7), Epstein–Barr virus (EBV), and human immunodeficiency virus (HIV), as well as various bacteria that interact with HPV and the human microbiome, increasing the risk of precancerous cervical cells leading to cancer [7,8].

## 2. The Human Microbiome and Its Impact on Viral Infections

### 2.1. Introduction to the Microbiome

The term “microbiome” describes a specific microbiota’s habitat and surrounding conditions, along with their collective genomes [9]. The human microbiome is defined as the genomic aggregate of organisms (microbiota) inhabiting various anatomical body sites, such as the skin, mucosa, gastrointestinal tract, respiratory tract, urogenital tract, and mammary gland. There are approximately 4 × 10^13^ bacteria in the human gastrointestinal tract alone, which is slightly more than all of our own cells together [10]. The combination of the host genome (about 20,000 genes in humans) and related collective microbial genomes (more than 33 million genes) is referred to as the hologenome (holobiont genome) [11].

The human microbiota forms a unique ecosystem that adjusts to the environmental conditions of the above-mentioned niches in a way that the general health and wellbeing are maintained, leading to a steady symbiosis. However, an unbalanced microbiota (dysbiosis) can lead to life-threatening health conditions, including cancer, cardiovascular disease, bowel inflammatory disease, and difficult-to-treat bacterial infections due to antibiotic resistance [12]. The largest concentration of the human microbiome is found in the gut, which plays a crucial role in maintaining human homeostasis. The National Institutes of Health launched the Human Microbiome Project (HMP) in 2007 to characterize and comprehend the role of the microbiome in human health and disease [13]. The HMP revealed that shifts in the host immune responses cause dysbiotic flora in the gut [14,15].

### 2.2. Microbiome Composition

Whipps et al. first defined microbiome as “a characteristic microbial community occupying a reasonably well-defined habitat which has distinct physio-chemical properties” [16], and to date, this term has been enriched by the numerous microbial functions and properties that form the ecological niches [17]. The microbiome refers to microbial assembly in a specific environment, along with their genetic information and functions, known as the metagenomic element of the microbiota [18]. Microbiomes from different bodily regions (digestive tract, respiratory tract, reproductive tract, etc.) collectively form a microecological system with the host. This system significantly influences human body part development, with alterations in the microecology leading to abnormal development and disease [19]. Both host and microbial genomes act in synergy to confer distinct phenotypes in humans. Therefore, understanding microbial populations within our body and their functions is of extreme clinical significance.

Traditionally, microbiome studies used culture-based methods to identify bacterial species. Over the last two decades, the introduction of next-generation sequencing (NGS) technology has enhanced the diagnostic and prognostic efficacy of microbial analysis. Undoubtedly, culture-based methods are still informative, but they detect only a restricted and known proportion of organisms that are not representative of every ecological niche. Similarly, optical magnification techniques have also been used to identify bacteria based on phenotype or morphological details, but today, the preferred methods for investigation are sequencing technologies with taxonomy-associated marker genes, such as the 16S rRNA or whole-genome sequences [20,21]. The whole-genome sequencing (WGS) approach is a high-throughput method through which all the bacterial genomes are studied in order to reveal the function of the genes and to identify novel genes and the pathways they are involved in [22].

Microbial assembly is highly dynamic, driven by host genotype, inter-microbial interactions, and environmental factors like lifestyle choices. Such community assembly passes through four major ecological and evolutionary processes, namely, dispersal, selection, diversification, and drift. In detail, dispersal refers to the immigration and emigration of microbes across space and time. The meta-community represents the set of local microbial species present across all habitats. Various factors, such as motility and distance from the source community, can limit microbial dispersal between local habitats [23]. Selection is an evolutionary process wherein better adapted species tend to survive better within their community and reproduce while displacing the poorly adapted species. Selective pressures in the form of habitat filters, including pH, oxygen level, resource availability, immune system state, and inter-microbial competition, may induce either a positive or a negative selection. Diversification refers to the generation of novel genetic variants within a population. Expansion and diversification of the human microbiome occur in a niche-specific manner from early life to senior years. Drift is defined by stochastic alterations in species abundances, which can disproportionately impact low-abundance species, leading to a potential extinction [24,25].

The first extensive application of NGS in microbiome study was the HMP, which included a total of 4788 specimens from 300 healthy individuals. A combination of organismal and functional data across body habitats was collected, providing an initial characterization of the normal microbiota in healthy adults. As disclosed therein, microbial carriage varied widely while metabolic pathways remained stable within healthy subjects [26].

### 2.3. Genital Microbiome

The human vaginal microbiota plays a crucial role in maintaining vaginal health. The human vagina is inhabited by a plurality of bacteria serving as a first line of defense against vaginal infections. Disruptions of normal vaginal microbiota have been associated with pelvic inflammatory disease, fertility issues, and adverse obstetric outcomes [27]. The vagina is a fibromuscular structure made up of three main layers, namely mucosa, muscle, and adventitia. The vaginal mucosa is composed of stratified squamous nonkeratinized epithelium, acquiring oxygen, glucose, and other nutrients from underlying submucosal tissues, creating an anaerobic habitat. The diversity and balance within the vaginal microbial communities may determine whether bacterial vaginosis, yeast infections, sexually transmitted diseases (STDs), and/or urinary tract infections emerge and persist [28].

**Figure 1:**
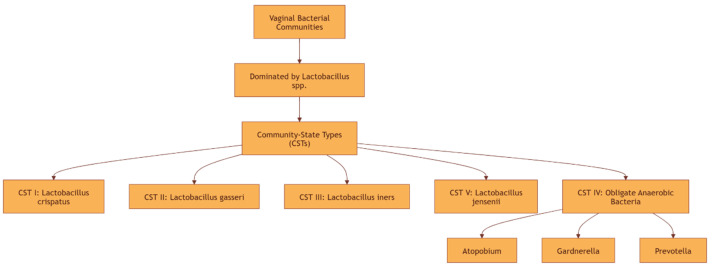
Vaginal microbiome classification.

Vaginal microbiota composition varies among women due to multiple factors, such as hormonal profiling, sexual activity, hygiene practices, and antibiotic and contraceptive use [29]. Despite composition variations, vaginal microbiota is dominated by *Lactobacillus* spp., which are considered beneficial microbes [30]. Indeed, vaginal bacterial communities cluster into three to nine discrete groups, most of which are dominated by *Lactobacillus* spp., while others include a combination of facultative and obligate anaerobes [14,31,32,33]. As disclosed by Ravel et al. [34], five community-state types (CSTs) exist in the vaginal microbiome, with *Lactobacillus crispatus*, *Lactobacillus gasseri*, *Lactobacillus iners*, and *Lactobacillus jensenii* dominating CSTs I, II, III, and V, respectively, and obligate anaerobic bacteria (*Atopobium*, *Gardnerella*, *Prevotella* spp.) dominating CST IV (Figure 1). Another classifier tool was recently published by France et al., namely VALENCIA (vaginal community state type nearest centroid classifier), for standardized assignment of CSTs within the vaginal microbiota of reproductive age women. Seven CSTs were identified, four of which had a high relative abundance of *Lactobacillus* spp. In particular, CST I was dominated by *L. crispatus*, CST II by *L. gasseri*, CST III by *L. iners*, and CST V by *L. jensenii*. The other three CSTs, termed CST IV-A, IV-B, and IV-C, had a low relative abundance of *Lactobacillus* spp. CST IV-A was enriched with *Candidatus lachnocurva vaginae*, but with a low relative abundance of *Gardnerella vaginalis*, while CST IV-B was dominated by *Gardnerella vaginalis*, with a low relative abundance of *Candidatus lachnocurva vaginae*. *Atopobium vaginae* was present in relatively low abundances in both CST IV-A and IV-B. CST IV-C was characterized by a low relative abundance of *Lactobacillus* spp., *Gardnerella vaginalis*, *Atopobium vaginae*, and *Candidatus lachnocurva vaginae*; instead, a wide variety of facultative and strictly anaerobic bacteria were present [35].

On the other hand, microbiota in the male reproductive system is extensively less studied, yet should not be underestimated, since it has important implications for male reproductive health, men’s fertility, and sexual behavior. Studies investigating medical male circumcision have provided the strongest evidence to date for the possible involvement of the penile (glans, coronal sulcus, foreskin, and shaft) microbiota in dysbiosis-related disorders, including sexually transmitted infections (STIs) [36,37].

The various surfaces of the penis, including glans, corona, urethra, corpora cavernosa, corpus spongiosum, and prepuce, each reflect distinct microenvironments, with varying moisture, oxygen availability, and keratinization, that serve as suitable habitats for unique microbial communities. In the absence of epithelial lesions, the keratinized squamous epithelium layer covering the outer foreskin is generally impermeable to STIs. Immunological cells found in the penile mucosal epithelium either prevent or induce infections by pathogens. Therefore, changes to the penis’s physical and immune system are likely to have an impact on bacterial colonization [38,39].

The human penile microbiome consists of various aerobic, anaerobic, facultative anaerobic, and microaerophilic bacteria, including *Prevotella*, *Peptoniphilus*, *Porphyromonas*, *Finegoldia*, *Corynebacterium*, *Anaerococcus*, *Staphylococcus*, and *Dialister* [40,41,42]. As demonstrated, circumcision can significantly affect penile microbiota. The abundance of putative anaerobic genera, including *Prevotella*, *Anaerococcus*, *Finegoldia*, and *Peptoniphilus*, has been reported to reduce considerably upon increased exposure to aerobic conditions, with an increase in *Corynebacterium* spp. and *Staphylococcus* spp., resulting in less diverse bacterial communities [39,43]. Another study revealed higher relative abundances of *Porphyromonas* and *Prevotella* and lower relative abundances of *Staphylococcus* in uncircumcised adolescent men compared to circumcised ones. On the other hand, common bacteria, such as *Corynebacterium*, *Finegoldia*, *Gardnerella*, and *Anaerococcus*, differ in relative abundance depending on the circumcision status and/or penile site sampled [44]. The inhabitant penile bacterial communities can also be manifested by condomless sexual intercourse. Indeed, a ~2–10-fold increase in the relative abundances of *Corynebacterium*, *Lactobacillus*, *Pelomonas*, *Ralstonia*, and *Mycobacterium,* along with a 3–142-fold decrease in the relative abundances of *Dialister*, *Megasphaera*, *Shuttleworthia*, *Atopobium*, and *Prevotella,* was recently reported [45].

### 2.4. Oral Microbiome

The oral cavity plays a fundamental role in maintaining oral as well as systemic health, since the second-largest and most diverse microbiota after the gut is found therein, harboring at least 200–500 unique bacterial species with abundances of more than 20 million individual cells. The oral microbiome is defined as the assembly of microorganisms that reside in the human oral cavity. The anatomical sites in which bacteria can colonize the oral cavity are teeth, tongue, cheeks, gingiva, tonsils, hard palate, and soft palate, while in the dental enamel, bacterial biofilms are formed. The environmental conditions within the oral cavity are optimal for the growth and maintenance of colonized bacteria with an average temperature of 37 °C and a stable pH of 6.5–7 [46,47,48].

The human oral microbiome is a highly complex ecosystem, consisting of bacteria, microeukaryotes, archaea, and viruses. The oral cavity of the newborn is considered sterile, with bacterial colonization beginning at and shortly after birth, usually at the first feeding and onward. By the first year, the mouth is inoculated with the pioneer species, mainly by aerobes, including *Streptococcus salivarius*, *Lactobacillus*, *Actinomyces*, *Neisseria*, and *Veillonella* [49]. In adults, more than 600 species of bacteria harbor the oral cavity, including mainly the phyla, Firmicutes, Bacteroidetes, Proteobacteria, Actinobacteria, Spirochaete, Fusobacteria, Euryarchaeota, Chlamydia, Synergistetes, and Tenericutes [46]. With the development of NGS technology, oral fungi, the candidate phyla radiation (CPR) group, and viruses have also been identified as oral microbiome components [50]. Particularly for the CPR group, which constitutes a unique bacterial division, these CPR members are thought to influence the oral microbial communities in a parasitic or symbiotic manner [51].

Salivary flow selective forces create favorable biophysical gradients of moisture and pH for the colonized bacteria, release or clear food metabolites, and stimulate mucin secretion, resulting in taxa enrichment [52]. Upon hyposalivation, dysbiotic communities can emerge in the oral cavity, inducing microbe-mediated diseases, such as periodontitis and caries [53,54]. Periodontal disease-specific species, such as *Porphyromonas gingivalis*, *Tannerella forsythia*, and *Treponema denticola*, have not been detected in any sites of healthy oral cavities. Moreover, bacteria-mediated dental caries and deep dentin cavities, including *Streptococcus mutans*, *Lactobacillus* spp., *Bifidobacterium* spp., and *Atopobium* spp., have not been found in the supra- and subgingival plaques of healthy human teeth [55].

Basal immune system activation is crucial for eliminating any suspicious pathogenic agent because the oral cavity is exposed to a variety of infections. The well-known IL-17 pathway plays a crucial role in mediating mucosal surveillance and barrier integrity in the direction of immune protection [56,57,58]. Among the viruses found in the oral cavity, HPV has been linked to head and neck squamous cell carcinoma and is implicated in several oral diseases, such as papilloma, condylomas, and chronic skin or mucosal epithelial infections. HPV types 6, 11, and 16 are commonly associated with oral papillomas, while HPV type 2 is mostly responsible for benign warts [59].

## 3. Interaction with Viruses

### 3.1. Microbiome Impact on Viral Infections

A growing amount of evidence suggests that viral immunity could be impaired if the balance between the host immune system and the human microbiota is disrupted. HPV, HIV, influenza viruses, SARS-CoV-2, viral gastroenteritis, viral hepatitis, and viral upper respiratory tract infections (URTIs) can all be prevented or treated through host commensal organisms and microbiome therapies. The human microbiome, invasive viruses, and host physiology interact in a complex way; however, there is increasing evidence that the microbiome can affect the progression of viral diseases [60]. The commensal and probiotic organisms can both regulate and be regulated by invasive viruses through several mechanisms, thereby enabling them to either stimulate or suppress viral infections [61]. The mucus layer, innate immune defenses, and adaptive immune defenses are the three main lines of defense that viruses must overcome when they interact with mucosal surfaces, including the oral, vaginal, gastrointestinal, or respiratory environment [62].

### 3.2. Viral Infection Outcome Modification Mechanisms

The microbiome modulates the structure and function of the mucus layer, making the mucosal epithelium an essential defense barrier against viral infections [63]. The mucus layer serves as a filter, blocking microbial access to the epithelial layer while providing nutrients to the microbiota residing within and around it [64]. The mucous layer covering the epithelial surfaces, such as the GI tract, vagina, and lung, contains glycoproteins known as mucins, which serve as a physical barrier between the host epithelial cells and invasive pathogens. As demonstrated, porcine gastric mucins prevented infection of epithelial cells by small viruses, such as HPV type 16, Merkel cell polyoma virus, and a strain of influenza A virus [65]. Regarding the vaginal microbiota, unlike an *L. iners*- or *Gardnerella vaginalis*-dominant microbiota, an *L*. *crispatus*-dominant microbiota has been demonstrated to prevent the spread of HIV-1 virions [66].

Understanding the interplay between the microbiome and viral infections is of great research interest, since it reveals new pathways to treat viral infections, thus improving the efficacy of antiviral therapies [67]. The advent of metagenomics has contributed to the acknowledgment of viral diversity and interaction within the human microbiome [68]. The microbiome is considered a determinative factor in preventing and/or reducing the impact of various pathogenic viruses. One microbiome-mediated mechanism refers to enhancing host immunity and reducing the rates of virus replication and transmission.

Depending on microbiome composition, the severity of a viral infection may be differentiated. A recent study [69] analyzed human microbiomes before posing a norovirus challenge to them. The prechallenged microbiome of symptomatic subjects was compared to that of asymptomatic ones. Interestingly, the microbiomes of asymptomatic individuals were abundant in Bacteroidetes spp., while lacking Clostridia spp. Moreover, by modifying the environment within the host, the microbiome can interact with it to increase infection [70]. When it comes to pathogenic viruses, certain microbiomes are linked to increased susceptibility to infection. Surprisingly, women with microbiomes enriched with *Mycoplasma* spp., *Prevotella bivia*, *Prevotella melaninogenica*, *Sneathia sanguinegens*, and *Veillonella montpellierensis* had an increased risk of HIV acquisition [71]. Additionally, Oh et al. discovered a positive correlation between the high prevalence of *Atopobium vaginae* in the cervical microbiome and the incidence of cervical intraepithelial neoplasia in HPV-infected individuals [72].

Another mechanism refers to microbiome-mediated viral attachment to host cells. An example reveals that *Haemophilus influenzae* pretreatment enhances the susceptibility of bronchial epithelial cells to viral replication and inflammatory response to respiratory syncytial virus (RSV), mainly though upregulation of the intercellular adhesion molecule 1 (ICAM-1) expression [73]. On the contrary, microbial communities may inhibit viral attachment to host epithelial cells. Indeed, Su et al. demonstrated that the CD4 receptor detected on the surface of *Lactobacillus* spp. may enable capturing HIV-1, thereby blocking viral transmission in CD4 + cells [74].

Apart from modifying the viral infection outcome, the microbiome is revealed to drive virus progression. Ford et al. [75] demonstrated that a bacterial pathogen’s evolution toward reduced virulence was affected by the presence of *Enterococcus faecalis* in the host microbiome. Another mechanism arises via certain bacteria, such as Bacillus species, that produce virucidal antimicrobial peptides (AMPs) called bacteriocins. Such compounds act either by displaying antiviral activity before viral entry into human cells or by reducing cytopathic effects and viral release yield [76].

### 3.3. Metabolite-Mediated Modification Mechanisms

Other mechanisms by which the microbiome modulates host immunity have also been discussed. Microbial colonization exerts durable effects on immune function via secondary metabolites, foreign molecular patterns, and antigens [77]. The production of microbiota metabolites results in either beneficial or harmful immune responses for the host’s health. Such metabolites include small metabolic byproducts, such as short-chain fatty acids (SCFAs), and/or macromolecules, such as peptidoglycan and lipopolysaccharides (LPS). Microbiome-originated metabolites have a considerable impact on both the innate and adaptive immune system. Various Toll-like receptors (TLRs) are found in intestinal epithelial cells, including TLR2 and TLR4, which are associated with innate immunity. The intestinal epithelium serves as a key interface between the innate immune system and the intestinal microbiome [78]. Short-chain fatty acids have multiple potential beneficial effects on the host’s immune system. The microbiome produces three main SCFAs: butyrate, propionate, and acetate. The majority of SCFAs are beneficial to host health; however, butyrate is the main SCFA that affects the immune system. The main producers of butyrate are *Faecalibacterium parasitizes, Clostridium leptum, Eubacterium rectale*, and species of the *Roseburia* genus [79]. It is possible for both pathogenic and commensal bacteria in the intestine to produce a range of molecules that pattern recognition receptors (PRRs) can identify. Bacterial LPS, toxins, peptidoglycan, lipoteichoic acid (LTA), and flagellin are a few examples of these microbe- or pathogen-associated molecular patterns (MAMPs and PAMPs). PRR and MAMP interactions through signaling pathways and expression modifications induce the release of interferons and cytokines that modulate the immune system [80].

## 4. The Genital Microbiome and HPV

### 4.1. Microbiome Profiles in HPV Infection

The most common sexually transmitted infection is HPV, which is greatly linked to cervical cancer. However, most HPV infections are cleared and do not result in cervical cancer [81,82]. As disclosed in several studies, a host antiviral immune response is responsible for clearing HPV infections [83,84,85]. Growing evidence supports the vaginal microbiome’s regulatory role in the local host immune responses. Diseased states have been linked to increased diversity of the *Lactobacillus*-nondominated vaginal microbiome, whereas vaginal health has been linked to decreased diversity of the *Lactobacillus*-dominated vaginal microbiome [86]. Squamous intra-epithelial lesions (SIL) and cervical HPV have been linked to vaginal dysbiosis [87]. High-risk HPV or dysplasia/cancer are more common in vaginal microbiomes dominated by *L. iners* or non-*Lactobacilli* spp. than in vaginal microbiomes dominated by *L*. *crispatus* [88]. Bacterial vaginosis also affects the establishment and persistence of HPV. As observed, bacterial vaginosis in CST IV is associated with persistent HPV, and the related biomarkers are *Atopobium* spp. and the sialidase gene of *Gardnerella vaginalis* [89].

Moscicki et al. [85] revealed that inflammatory expression levels in women without HPV infection were comparable to those in women with HPV infection at the post-clearance visit. The local environment of women who had HPV16 differed from that of women who seemed to be protected from acquisition. The microbiome states of women with HPV 16 were comparatively unstable, with a high likelihood of fluctuating between states. Conversely, women with no history of HPV exhibited consistent states during the visits, mainly detected with *L. crispatus* and *L. iners*, which are both characterized as healthier states than non-*Lactobacillus* microbiota. Moreover, slight changes occurred over HPV persistence, with non-*Lactobacillus* emerging and *L. crispatus* initially increasing. As expected, *Gardnerella vaginalis* increased during HPV persistence and post-clearance since it is frequently found in non-*Lactobacillus* environments. Other studies disclosed that *L. gasseri*, *L. iners*, and anaerobic species, such as *Gardnerella vaginalis*, were also often found in women with HPV infection and high-grade intraepithelial lesions (HSIL) [90,91].

Additionally, the abundance of certain bacteria varies significantly among the various HPV types, especially for species belonging to the *Lacticaseibacillus*, *Megasphaera*, and *Sneathia* genera. Regarding *Lacticaseibacillus*, a significant reduction was observed in HPV16 and HPV18 cases compared to other high-risk HPV cases. Therefore, it is indicated that the observed severity of high-risk HPV infection, especially in the case of HPV16 and HPV18 types, may be related to the abundance of certain cervicovaginal microbial genera [92].

### 4.2. Impact on HPV Persistence and Clearance

Vaginal *Lactobacillus* spp. is crucial for maintaining the integrity of the cervical epithelial barrier, since it can prevent HPV from entering basal keratinocytes via bacteriocin production and low pH maintenance [93]. On the contrary, in women with both intermediate flora and bacterial vaginosis, a significant risk for persistent HPV and delayed rate clearance is observed [94,95]. HPV-positive women are found with a lower proportion of protective *Lactobacillus* spp. compared to HPV-negative women, while in women in whom *L. gasseri* is dominant, HPV clearance rates are increased [96]. Vaginal microbiota enriched in *L. iners* are more commonly related to lower HPV clearance rates. *L. iners* can survive in multiple environmental and metabolic stress-related conditions, and it does not efficiently prevent pathogen colonization. Moreover, through inerolysin, a cholesterol-dependent pore-forming cytotoxin, it opens a channel in the vaginal epithelium and facilitates pathogen entrance [97,98].

*Atopobium, Prevotella, Parvimonas, Gardnerella, Megasphera, Ruminococcaceae, Mobiluncus,* and *Sneathia* are commonly linked to the development of premalignant and invasive cervical cancer upon HPV infection, while *L. crispatus* and *L. gasseri* were recognized to be the most common species in women who tested negative for HPV [96,99,100]. According to a recent study, there was a significant correlation between HPV persistence and high proportions of *Gardnerella*, *Prevotella*, *Megasphoera*, and *Atopobium* [89]. A recent study revealed that among HPV-positive patients, the most common bacteria were *Gardnerella vaginalis*, *Enterococcus* spp., *Staphylococcus* spp., *Proteus* spp., and *Atopobium* [101]. Additionally, it was demonstrated that *Sneathia* spp. was the most prevalent bacteria in women with HPV infection and premalignant lesions, while *Fusobacterium* spp. was linked to cervical cancer [102].

Regarding the association between penile microbiota and HPV infection, researchers’ interest has been triggered by randomized control trials investigating the risk of HPV acquisition and medical male circumcision, through which glans thickening is achieved, thereby making it less susceptible to HPV [103,104,105]. A reduction in the risk of high-risk HPV infection is revealed, mainly through a decrease in local immune inflammation in the penile tissues that prevents loss of epithelial barrier integrity [106,107,108]. HPV infection is more common in the coronal sulcus in uncircumcised men, indicating that the moist subpreputial surface serves as a favorable environment for HPV acquisition [109]. A recent meta-analysis revealed that male circumcision reduced the prevalence of genital HPV infection by an average of 32% [110]. Additionally, a higher prevalence and lower HPV clearance rates have been reported in the glans/corona of uncircumcised men compared to circumcised ones [111]. The most recent additional evidence on the association between male circumcision and HPV infection disclosed a reduced incidence rate of HPV infection with increased HPV clearance at the glans penis, along with reduced prevalent infections on the shaft in circumcised men [112].

Male circumcision has been associated with changes in penile microbiota and reduced risk of HPV, including high-risk HPV and multiple HPV infections [113]. *Corynebacterium*-dominated penile microbiota have been linked to a reduced risk of high-risk HPV compared to non-*Corynebacterium*-dominated penile microbiota, including those dominated by bacterial vaginosis-associated bacteria or *Lactobacillus*. In particular, *Prevotella*-, *Clostridiales*-, and *Porphyromonas*-dominant penile microbiota are more frequent in HPV-infected men than in men with *Corynebacterium*-dominated penile microbiota. High-risk HPV infections are specifically linked to lower relative abundances of *Corynebacterium* and higher relative abundances of bacterial vaginosis-associated bacteria, including *Prevotella*, *Peptinophilus*, and *Dialister*, highlighting a potential protective role of *Corynebacterium* against HPV infection in men [114]. The most recent study in the penile microbiome was conducted in HPV-associated penile squamous cell carcinoma. It described the first microbiome of penile carcinoma, revealing abundant and diverse microbiota along with inflammatory-related taxa, including *Proteobacteria* and *Firmicutes*, *Fusobacterium* and *Prevotella*, and *Finegoldia magma* and *Pseudomonas geniculata* [115].

The exact mechanisms by which the penile microbiota facilitate or prevent HPV acquisition remain unclear. However, it has been suggested that anaerobic bacteria-dominant penile microbiota may modulate host immunity to HPV infection by altering the local immune environment of the penile skin. HPV-positive men with diverse microbiota could exhibit elevated levels of chemokines compared to men with *Corynebacterium*-dominated microbiota, thus triggering the activation of the immune system [114].

### 4.3. Influence on Cervical Cancer Development

It is well known that most HPV infections resolve within 2 years; however, HPV infections that persist are at risk of developing cervical lesions, mainly cervical intraepithelial neoplasia and cervical adenocarcinoma [116,117]. HPV infection alone is not sufficient to cause development. Mucosal surface-specific elements like immune regulation, mucosal secretions, epithelial surface integrity, and the local microbiota probably contribute to HPV persistence and cancer progression [118].

The cervicovaginal microbiota contributes significantly to the virus’s persistence and regression, which in turn has major implications for disease progression. A higher prevalence and persistence of HPV infection have been linked to a dysbiotic or highly diverse vaginal microbiota coupled with chronic subclinical inflammation [93]. Dysbiotic microbiota can either directly cause tissue damage, which makes it easier for oncoviruses to infect the host, or modify host mechanisms, such as enabling immune response modifications and DNA damage, which ultimately cause carcinogenesis [119].

The proinflammatory transcription factor nuclear factor-kB (NF-kB), tumor necrosis factor α (TNF α), IL-6, IL-8, and macrophage inflammatory protein 3α (MIP 3α) can be activated by certain bacteria, including *Atopobium* [89]. *Gardnerella vaginalis*, *Fusobacterium*, and *Sneathia* are additional bacteria that release the sialidase enzyme and break down mucus, thus making the cervical epithelium more susceptible to viral infection [120,121]. Moreover, *Fusobacterium* spp., via its virulence factor, Fad A, activates the WNT signaling pathway, which is an essential survival and proliferation pathway in cervical cancer [122].

Specific microbes, such as *Fusobacterium* spp., *Peptostreptococcus* spp., *Campylobacter* spp., and *Haemophilus* spp., are exclusively detected in cervical adenocarcinoma cases; therefore, such microorganisms could be considered potential biomarkers for cervical cancer development [123,124,125]. *Methylobacterium* spp. may be suggestive in HPV-negative subjects, whereas *Alloscardovia* spp., *Eubacterium* spp., and *Mycoplasma* spp. have been found to be potential biomarkers in HPV-positive ones [126,127,128,129].

## 5. Mechanisms of Microbiome Influence in HPV Infection

### 5.1. Immune Modulation

Dysbiosis can cause several cancer features, such as barrier disruption, excessive cellular proliferation, genetic instability, angiogenesis, chronic inflammation, and metabolic dysregulation. Dysbiosis-induced oxidative stress produces reactive oxygen species (ROS), which can damage proteins and lipids and cause double-stranded DNA breaks in the host genome and HPV episome, enabling HPV genome integration and, ultimately, cell transformation [93,99,130].

A complex barrier system consisting of an intact epithelium with tight junctions, secretion of soluble immune mediators, and a mucus layer protects the vaginal environment from HPV infection. Upon disruption of this barrier system, pathogenic microbes may move across the vaginal epithelia, causing low-grade chronic inflammation and other conditions, such as cancer [131]. The local vaginal microbiota enables the modulation of immune responses to HPV infection. Indeed, certain microbial communities can activate immune cells, regulate adherence junction proteins, and modulate inflammation, thus affecting HPV clearance or persistence. *L. iners*, *Gardnerella*, *Prevotella*, and *Megasphaera* are indicated as HPV persistence-related species, while *L. crispatus* exerts a protective effect. Proinflammatory cytokines, including IL-1β and TNF-α, are increased in the presence of anaerobic bacteria, such as *Prevotella*, *Dialister*, *Atopobium vaginae*, *Sneathia*, *Adlercreutzia*, *Peptoniphilus*, and *Megashpaera*, and inversely correlated with *Lactobacillus* dominance [132,133,134].

The protective role of *Lactobacillus* during HPV entrance is supported by lactic acid, bacteriocins, polysaccharides, peptidoglycans, and hydrogen peroxide (H_2_O_2_) production, thus reducing pH, enhancing the viscosity of cervicovaginal mucus, and preventing the adhesion of cells to epithelial tissue. The above-mentioned Lactobacillus byproducts exert beneficial effects by either modulating inflammatory immune responses or inducing acquired immune responses [135]. On the other hand, certain vaginal microbiota, such as *Gardnerella vaginalis*, may modulate host immune responses, including a shift from antimicrobial to antiviral responses in the female genital tract [136].

### 5.2. Microbial Metabolites

Individuals with and without HPV infection present with distinct metabolomic profiles. As recently observed, HPV-positive women had increased levels of biogenic amines and glycogen-related metabolites in *L. iners*-enriched microbiota and decreased levels of glutathione, glycogen, and phospholipid-related metabolites in microbiota, with an abundance of *Atopobium*, *Prevotella*, *Parvimonas*, *Gardnerella*, *Megasphera*, *Ruminococcaceae*, *Mobiluncus*, and *Sneathia* and lack of *Lactobacillus* spp., compared to HPV-negative ones [137]. Additionally, high levels of three lipid compounds, including 3-hydroxybutyrate, eicosenoate, and oleate/vaccinate, were detected in women with cervical cancer [99,138].

Vaginolysin, a cholesterol-dependent cytotoxic protein, is secreted mainly from *Gardnerella vaginalis*-enriched microbiota, followed by *L. iners* microbial community. It may contribute to bacterial vaginosis and induce tissue damage and cellular lysis [139]. A metabolic byproduct of *Lactobacilli*, lactic acid, contributes to pH maintenance, inhibits the growth of pathogens, and enhances the local immune system. This acidification of the vaginal surface creates a protective barrier against HPV infection [140]. Women with *L. iners*-dominant microbiota and *Atopobium*-, *Prevotella*-, *Parvimonas*-, *Gardnerella*-, *Megasphera*-, *Ruminococcaceae*-, *Mobiluncus*-, and *Sneathia*-dominant microbiota present a greater L-to-D-lactic acid ratio, which enhances the production of extracellular matrix metalloproteinase inducer (EMMPRIN), activating matrix metalloproteinase (MMP-8). By cleaving collagen, MMP-8 breaks down intracellular junctions, modifies cervical integrity, and enables HPV to enter basal keratinocytes. Additionally, MMP-8 and EMMPRIN are related to cancer progression [66,141]. Moreover, novel metabolites of 9,10-DiHOME, α-linolenic acid, ethylparaben, glycocholic acid, pipecolic acid, and 9,12,13-trihydroxy-10(E),15(Z)-octadecadienoic acid correlating with *Sneathia amnii*, *L*. *iners*, *Atopobium*, *Mycoplasma*, and *Gardnerella*, which could serve as potential biomarkers of HPV infection, were revealed [142]. The most recent data suggest that N-methylalanine, phenylacetaldehyde, succinic acid, 2-3-dihydroxypyridine, DL-p-hydroxylphenyllactic acid, gluconic acid lactone, guanine, glucose-6-phosphate, erythrose, and sucrose have significant associations with HPV-induced cervical lesions [143].

### 5.3. Microbial Competition

Microbial communities inhabit extremely competitive environments within the vaginal ecosystem, with their survival and persistence depending on prevailing environmental conditions. A suitable vaginal microenvironment that prevents HPV infection involves maintaining an acidic pH (<4.5) and Lactobacillus dominance that, via hydrogen peroxide, organic acids, and bacteriocins, provide the vaginal ecosystem stability. Therefore, vaginal microbiota enriched in *Lactobacillus* spp. can prevent pathogens from adhering to the vaginal epithelium or colonizing it, thus protecting against HPV infection and other viruses [144,145,146]. *Lactobacillus* can enable cancer cell apoptosis, inhibit cancer cell proliferation, and regulate genes involved in metastasis, thus preventing the occurrence and progression of cervical cancer. Direct and indirect *Lactobacillus*-mediated mechanisms can hamper cervical cancer progression. The E6 and E7 oncoproteins are the main mechanisms by which high-risk HPV interferes with regular cell cycle checkpoints, enhances uncontrolled cell proliferation, and inhibits apoptosis. The HPV genome frequently integrates into the host cell genome during carcinogenesis, resulting in E2 gene disruption, overexpression of the E6 and E7 oncogenic proteins, and cellular transformation. Accordingly, various host gene modifications can occur, including point mutations, chromosomal abnormalities, and/or altered methylation patterns, which enable cervical intraepithelial neoplasia to evolve to cervical cancer. The indirect mechanism of *Lactobacillus* to prevent cervical carcinogenesis involves the downregulation of HPV oncogene expression [135,147].

**Figure 2:**
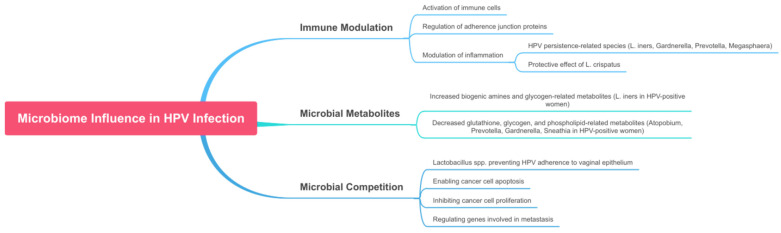
Mechanisms of microbiome influence in HPV infection.

Regarding *L. crispatus*, it was demonstrated that it induced a substantial reduction in biogenic amine levels, preventing recurrent inflammation and maintaining the homeostasis of the vaginal microbiota [148]. The beneficial effect of *L. crispatus* has also recently been highlighted. Therein, healthy women exhibited a higher relative abundance of *L. crispatus* compared to women with cervical dysplasia that had a substantial vaginal microbial diversity with higher abundances of *Gardnerella vaginalis*, *Aerococcus christensenii*, *Peptoniphilus lacrimalis*, and *Fannyhessea vaginae* [149]. The most recent data highlight the close association between vaginal microbiota and cervical dysplasia stages, characterized by a higher prevalence of pH  >  5, lower hydrogen peroxide levels, and vaginal microbiota lacking *Lactobacillus* spp., especially *L. crispatus*, while being enriched with more non-*Lactobacillus* spp., such as *Actinomyces* and *Burkholderiaceae* [150]. A graphical presentation of all the above-mentioned mechanisms of microbiome influence in HPV infection is provided in Figure 2.

A novel viral immune evasion mechanism was recently revealed, demonstrating that HPV suppresses the expression of host defense peptides that are activated by pro-inflammatory and basal factors [151]. Another pathway explaining long-term HPV persistence is via the downregulation of type I interferons, including IFN-α, IFN-β, and TLR3 [152]. An additional mechanism that emerged from studying the oral microbiome and HPV in terms of oropharyngeal carcinogenesis proposes HPV-microbial crosstalk as facilitating HPV virion entry to the basal keratinocytes and creating an immune environment suitable for HPV persistence [153].

## 6. Therapeutic Implications and Future Directions

### 6.1. Probiotics and Prebiotics in HPV Infection

A healthy vaginal environment, which is predominantly populated by lactobacilli, is known to serve a protective role against reproductive and STIs [154]. In contrast, dysbiosis confers to the increase, among other vaginal infections, in HPV infection risk and progression to cervical cancer [155,156].

Recent studies indicate that restoring a balanced vaginal microbiota, particularly with the use of probiotics and specifically with *lactobacillus* species, may help in disrupting the cycle of infection and promoting HPV clearance [157,158,159,160]. Strain-specific differences in *Lactobacillus* species should be taken into account, as they have been observed to have varied effects, highlighting the importance of selecting the appropriate probiotic strains for therapeutic use [161,162].

Early studies [163,164] have demonstrated that *Lactobacillus plantarum* and *Lactobacillus acidophilus* exhibit favorable probiotic properties and significant anticancer activity against human cancer cell lines, without causing cytotoxic effects on normal cells. These beneficial effects have also been confirmed by more recent studies. Regarding these strains, laboratory research has shown that *L. plantarum* and *L. acidophilus* not only prevent the growth of harmful bacteria that contribute to vaginal dysbiosis and cervical intraepithelial neoplasia (CIN) progression, but also secrete postbiotic compounds that suppress malignant cell growth and inhibit cancer progression [165].

Studies have associated *Lactobacillus gasseri* with the rapid elimination of newly acquired HPV infections [96]. Alongside *Lactobacillus crispatus*, this species has demonstrated selective cytotoxic effects against HPV18-infected HeLa cervical cancer cells, while having no impact on normal cervical cell lines. This effect appears to be independent of pH or lactate concentration, indicating a more intricate mechanism at play [166,167].

Furthermore, a semi-randomized interventional study involving HPV-positive women with low-grade cervical lesions found that those receiving oral Lactobacillus casei had higher rates of HPV clearance and were significantly more likely to resolve their cervical lesions compared to an untreated control group [168]. Additionally, *Lactobacillus casei* and *Lactobacillus paracasei* strains isolated from human breast milk have shown effectiveness against HeLa cells [169].

Palma et al. [170] explored the effects of long-term vaginal administration of *Lactobacillus rhamnosus* over short-term (three months) and long-term (six months) periods to restore vaginal microbiota in HPV-infected women. The long-term treatment group demonstrated a significantly higher resolution rate of HPV-related cytological abnormalities, with HPV clearance being more prevalent in this group. Consistently, other studies have indicated that *Lactobacillus rhamnosus, Lactobacillus crispatus,* and *Lactobacillus gasseri* exhibit cytotoxic effects on cervical tumor cells, while leaving normal cells unaffected [171,172].

Given the connection between intestinal and vaginal microbiota, oral probiotic supplementation also appears to be a promising strategy for restoring microbial balance in HPV-infected women [173,174,175]. A recent study examined the effects of oral supplementation with *Lactobacillus crispatus*, M247, a species known to colonize both the vaginal and intestinal microbiota in HPV-positive women [176]. The findings indicated a higher HPV clearance rate in the probiotic group compared to the control group. Oral intake of *Lactobacillus crispatus* enables its colonization in the intestine, forming a reservoir that naturally transfers to the vagina within days [177].

Similarly, among women with HPV-positive precancerous lesions, a six-month intervention involving a daily probiotic drink containing *Lactobacillus casei* strain Shirota led to a significantly higher clearance rate [168].

However, in another study that attempted to assess the impact of probiotics on genital high-risk HPV (hrHPV) infection, oral administration of *Lactobacillus rhamnosus* GR-1 and *Lactobacillus reuteri* RC-14 did not show a significant difference in clearance rates compared to the placebo group [178].

Prebiotics also seem to play a crucial role in lowering HPV positivity rates and reducing the incidence of low-grade cervical lesions. Certain prebiotics contribute to the restoration of cervical mucosal structure and promote the proper maturation of metaplastic epithelium, resulting in improved colposcopic outcomes [179]. Additionally, they create a protective mucoadhesive film over the cervical surface, acting as a barrier against harmful microbial agents. In this context, a large study found that a higher dietary fiber intake was associated with a reduced risk of HPV infection [180].

While further research is needed to clarify the exact role of vaginal microbiota in cervical disease progression, identify the most protective bacterial strains against HPV-induced dysplasia and neoplasia, and determine optimal therapeutic dosages, pre- and probiotic-based approaches may offer a viable and accessible strategy for reducing HPV-related disease burden.

### 6.2. Vaginal Microbiota Transplantation

Due to the similarities between the gut and the vagina in terms of physiological conditions and the development of infections caused by pathogen overgrowth, researchers have recently expanded the use of probiotic therapy and proposed vaginal microbiota transplantation (VMT) as a promising treatment for vaginal infections [181]. VMT involves transferring the entire vaginal microbiota community or specific probiotic strains with beneficial properties from the vaginal secretions of healthy donors to recipients, with the goal of establishing a restored vaginal micro-ecological balance. Given the established link between dysbiosis and human papillomavirus (HPV) infection, as well as cervical cancer (CC) progression, microbiome transplantation could offer a novel approach to enhancing HPV clearance and reducing the risk of malignant transformation [182,183,184,185]. Several mechanisms support this hypothesis, such as (a) restoration of protective microbiota, (b) promotion of local immune responses to improve viral clearance, (c) competitive exclusion of pathogens, and (d) production of postbiotics and metabolites with antiviral and anticancer properties [166,186].

Several studies have demonstrated the safety and efficacy of VMT in treating bacterial vaginosis [183,187,188], recurrent yeast infections [189], and other vaginal conditions [190] using methods that either involve the direct transplantation or inoculation of vaginal fluid obtained from a healthy individual or the direct transplantation of particularly cultured derivatives [191]. The procedure has also shown promising results in reducing the risk of sexually transmitted infections and preterm births in pregnant women [192].

While these mechanisms are promising, several challenges remain before microbiome transplantation can be considered a viable clinical approach, since the feasibility, efficacy, and ethical implications of this approach must be carefully evaluated [193].

Donor selection and screening pose a major obstacle, as an optimal microbial donor must be free from infectious diseases, sexually transmitted infections, and dysbiosis-related conditions. Unlike stool-based FMT, which has standardized protocols, VMT lacks established guidelines, making safety assessments more difficult. Additionally, the long-term effects of microbiome transplantation remain unknown, making it imperative to establish strict protocols for donor screening, microbial assessment, and patient monitoring before it can be considered a viable therapeutic option.

Beyond the physical risks, the ethical implications of informed consent and donor selection add another layer of complexity to microbiome transplantation. Since HPV-infected individuals, particularly those with CIN, may have compromised immune defenses, the introduction of an unregulated or inadequately screened microbial community could pose serious health risks. The absence of standardized donor criteria raises ethical concerns about ensuring patient safety while maintaining accessibility to treatment.

Regulatory oversight is another pressing issue, since the classification of microbiome-based therapies under existing medical and pharmaceutical laws remains unclear. Without clear guidelines, there is a risk that microbiome transplantation could be adopted in unregulated clinical settings, exposing patients to poorly screened or ineffective treatments. The social and psychological acceptability of microbiome transplantation must also be considered, since public perception of vaginal microbiota transfer may face additional cultural and ethical barriers.

### 6.3. Personalized Medicine

Personalized medicine can play a pivotal role in the management of HPV infection and the microbiome by tailoring prevention, diagnosis, and treatment strategies to an individual’s unique biology. Using advanced multi-omics technologies like metagenomics, transcriptomics, and metabolomics, a detailed assessment of microbial composition and host interactions can be achieved, allowing for microbiome interventions that are tailored to each patient’s specific microbial profile [194,195]. For HPV-infected individuals, this means that treatment strategies could be adapted based on dysbiosis patterns, immune responses, and genetic predispositions, optimizing effectiveness while minimizing risks.

In terms of diagnosis, biomarkers derived from vaginal fluids, cervicovaginal secretions, or blood can be utilized to detect HPV-related disease progression, providing a more tailored approach to monitoring infection status [194,196]. Current promising biomarkers for cervical precancer lesions and cervical cancer detection are HPV methylation markers. Indeed, HPV methylation has emerged as a considerable epigenetic molecular tool in cervical intra-epithelial neoplasia assessment, as higher HPV methylation rates are associated with increased disease severity [197]. Particularly for DNA methylation markers ASCL1 and LHX8, they were shown to yield a high sensitivity for cervical cancer detection, constituting a possible direct triage method for cancerous lesions in HPV-positive women [198]. Moreover, it was recently revealed that methylation positivity rates of other gene targets, FAM19A4 and hsa-miR124-2, were associated with high-grade squamous intraepithelial lesions and CIN cases, along with a persistent infection of high-risk HPV. Accordingly, methylation was detected in about 60% of CIN1 lesions and in 83.3% in CIN2/3 cases [199].

Another area where personalized medicine can be integrated is immunotherapy and vaccine strategies [200,201]. Mapping the interactions between the microbiome and the immune system can provide insights into how personalized immunotherapy could enhance the body’s response to HPV [202]. Similarly, understanding individual microbiome profiles may allow for more effective HPV vaccination strategies, potentially improving vaccine efficacy based on microbial composition [203]. HPV vaccination appears to have minimal impact on the composition of the vaginal microbiome. While minor fluctuations in bacterial abundance may occur, particularly in Lactobacillus species, no significant changes in microbial diversity have been observed in previous studies [204]. Additionally, slight decreases in both pro-inflammatory and anti-inflammatory cytokine levels suggest that the vaccine does not provoke substantial immune-driven alterations in the vaginal environment. However, further research with larger and more diverse populations is necessary to fully understand potential variations across different demographics and over extended periods.

Furthermore, artificial intelligence and big data can help analyze microbiome data [205], immune responses [206], and patient history to develop individualized treatment plans. Digital health tools could track changes in the microbiome and immune responses, enabling real-time adjustments to treatments [207]. Advanced deep learning models serve as powerful AI tools capable of analyzing microbiome data, identifying patterns, and predicting health outcomes, including potential disease markers and the effectiveness of probiotic treatments. These technologies facilitate a more comprehensive understanding of the microbiome’s role in human health, paving the way for AI-driven advancements in microbiome-based therapies. Additionally, AI-powered algorithms can enhance probiotic interventions by tailoring treatments to individual responses, considering genetic and immune profiles. This personalized strategy holds the potential to significantly enhance the effectiveness of probiotic therapies, minimize side effects, and optimize clinical results.

Artificial intelligence (AI) has the potential to revolutionize the analysis of the microbiome and enhance personalized treatment strategies for HPV-related cancers. AI-powered machine learning algorithms can process vast amounts of genomic, metagenomic, and clinical data to identify patterns in microbiome composition associated with HPV infection and cancer progression. By integrating microbiome profiling with patient-specific immune responses, AI can predict disease risk, assess treatment efficacy, and optimize therapeutic approaches. AI-driven models can also aid in early detection of HPV-related cancers by analyzing microbial biomarkers, allowing for timely interventions. Additionally, AI can support personalized treatment plans by identifying microbiome-targeted therapies, such as probiotics or immunomodulatory interventions, that may enhance vaccine efficacy or improve patient outcomes. Through advanced data analytics and precision medicine, AI can play a crucial role in transforming HPV-related disease management, leading to more effective prevention and treatment strategies.

## 7. Strengths and Limitations

The present review demonstrates several significant advantages. It provides a comprehensive and thorough description of the interactions between genital and oral microbiota and HPV infection and the mediated mechanisms via which HPV infection persists and progresses into cancer. Importantly, all the latest potential therapeutic approaches that emerge from leading-edge technologies, such as NGS and AI-based solutions, are discussed. Another distinct advantage is the description of the penile microbiota composition and its association with HPV infection, since microbial communities in the male reproductive system are generally less studied. The main limitation of our review is that it primarily examines HPV interactions within two specific microbial niches—the genital and oral environments—since HPV infection is associated with certain cancer types, particularly cervical cancer. Moreover, since we present a narrative review, a rigorous statistical analysis in terms of a corresponding meta-analysis is lacking; therefore, no stronger evidence supporting all the above-mentioned interactions can be provided. Furthermore, given the evolving nature of research on the HPV vaccine, microbiome, and immune interactions, there may be relevant studies that have not yet been published at the time of our review.

## 8. Conclusions and Future Directions

The intricate relationship between the human microbiome and HPV infection extends beyond the vaginal microbiota, encompassing the oral, gut, and other body site microbiomes. Emerging research highlights that microbial communities across these anatomical niches can influence immune responses, viral persistence, and disease progression. The oral microbiome, for instance, has been linked to HPV-related oropharyngeal cancers, where dysbiotic microbial environments may create conditions conducive to viral persistence and carcinogenesis. Similarly, the gut microbiome plays a crucial role in systemic immune modulation, potentially affecting the body’s ability to clear HPV infections.

The therapeutic potential of microbiome-targeted interventions, including probiotics, prebiotics, and vaginal microbiota transplantation, offers potential avenues for restoring microbial balance and enhancing HPV clearance. While vaginal microbiome modulation has shown promise in enhancing HPV clearance and reducing cervical cancer risk, modifying the gut and oral microbiomes could further optimize immune responses against HPV. However, challenges remain in standardizing these approaches, ensuring safety, and understanding the long-term implications of microbial interventions.

Personalized medicine, driven by advanced multi-omics technologies and artificial intelligence, holds significant promise for tailoring HPV management strategies based on individual microbiome profiles. By leveraging machine learning and big data analytics, researchers can develop predictive models for HPV progression and treatment response, ultimately improving patient outcomes.

Further research is needed to establish standardized clinical protocols for microbiome-based therapies and to clarify the long-term implications of interventions, such as VMT. As our understanding of the microbiome–HPV axis evolves, integrating microbiome-targeted approaches into clinical practice could revolutionize the prevention and treatment of HPV-related diseases, moving toward a more personalized and effective healthcare paradigm.
